# Creation of a High-Resolution, Continually Evolvable Lineage Tracer

**DOI:** 10.64898/2026.09.30.754670

**Published:** 2026-10-01

**Authors:** Ryan N. Delgado, Yichen Si, Rebecca E. Andersen, ChangHee Lee, Henry L. Bushnell, Jordan L. Doman, Smriti Pandey, Alexander A. Sousa, Emma R. West, Carina E. Dagotto, David R. Liu, Constance L. Cepko

## Abstract

Cellular diversity is required for the proper function of many tissues, including the retina, with its ∼130 cell types. ^1-3^ Previous lineage studies suggested that retinal progenitor cells (RPCs) generate cell diversity through biased cell divisions. ^4-9^ However, the extent to which each progenitor cell contributes to diversity, and the overarching rules governing the lineages of the collection of progenitor cells, are unknown. While classic lineage tracing approaches including retroviral labeling ^4,6,10^ , dye injection ^11,12^ , and time-lapse microscopy ^13,14^ were used to make the foundational insights regarding retinal lineages, they cannot be used to interrogate multigenerational lineages at scale. Here we describe the creation of an evolvable lineage tracing tool, “SCRIBBLE” for S equential C ombinatorial R ecorder for Iterative B arcode- B ased L ineage E volution. SCRIBBLE uses prime editor to iteratively edit a barcode sequence while preserving the order of genesis of clonally related progeny. Through a series of rationally designed high-throughput screens and design optimizations, we identified novel sequences that support a high level of barcode editing and information content. Evaluation of SCRIBBLE in embryonic mouse retina recapitulated known lineages in retinal development, demonstrating that SCRIBBLE is capable of high-resolution lineage tracing in a complex tissue.

## Full Text Availability

The license terms selected by the author(s) for this preprint version do not permit archiving in PMC. The full text is available from the preprint server.

